# In Silico Proteomic Profiling and Bioactive Peptide Potential of Rapeseed Meal

**DOI:** 10.3390/foods14142451

**Published:** 2025-07-12

**Authors:** Katarzyna Garbacz, Jacek Wawrzykowski, Michał Czelej, Adam Waśko

**Affiliations:** 1 Biolive Innovation Sp. z.o.o., Dobrzańskiego 3, 20-262 Lublin, Poland; 2 Department of Biotechnology, Microbiology and Human Nutrition, Faculty of Food Science and Biotechnology, University of Life Sciences in Lublin, Skromna 8, 20-704 Lublin, Poland; adam.wasko@up.lublin.pl; 3 Department of Biochemistry, Faculty of Veterinary Medicine, University of Life Sciences in Lublin, Akademicka 12, 20-033 Lublin, Poland

**Keywords:** rapeseed protein, peptide biological activity, in silico digestion

## Abstract

Rapeseed meal, a byproduct of oil extraction, is increasingly recognised as a valuable source of plant protein and health-promoting peptides. This study aimed to identify key proteins in cold-pressed rapeseed meal and assess their potential to release bioactive peptides through in silico hydrolysis using plant-derived proteases, namely papain, bromelain, and ficin. Proteomic profiling via two-dimensional electrophoresis and MALDI-TOF/TOF mass spectrometry revealed cruciferin as the dominant protein, along with other metabolic and defence-related proteins. In silico digestion of these sequences using the BIOPEP database generated thousands of peptide fragments, of which over 50% were predicted to exhibit bioactivities, including ACE and DPP-IV inhibition, as well as antioxidant, neuroprotective, and anticancer effects. Among the evaluated enzymes, bromelain exhibited the highest efficacy, yielding the greatest quantity and diversity of bioactive peptides. Notably, peptides with antihypertensive and antidiabetic properties were consistently identified across all of the protein and enzyme variants. Although certain rare functions, such as anticancer and antibacterial activities, were observed only in specific hydrolysates, their presence underscores the broader functional potential of peptides derived from rapeseed. These findings highlight the potential of rapeseed meal as a sustainable source of functional ingredients while emphasising the necessity for experimental validation to confirm the predicted bioactivities.

## 1. Introduction

Rapeseed (*Brassica napus* L., family Brassicaceae) is the second most extensively cultivated oilseed crop globally, following soybean, with an estimated production of 85.1 million tons in 2024/25 [1]. It is predominantly cultivated for its oil-rich seeds, which are integral to major industries, such as edible oil, food processing, and biodiesel production [2]. The European Union alone contributes approximately one-quarter of the global rapeseed production (16.86 million tons) [1], with Poland, France, and Germany being leading producers [2,3]. The process of extracting oil from rapeseeds results in rapeseed meal, a protein-rich co-product that constitutes nearly half of the seed’s mass (its typical composition includes approximately 40–50% oil, 15–30% protein, 20–30% fibre, and 5–10% moisture) [4]. Traditionally, rapeseed meal has been utilised as animal feed or fertiliser; however, there is increasing interest in valorising this abundant resource for food and nutraceutical applications. Rapeseed meal contains approximately 35–40% protein and has a well-balanced amino acid composition, rendering it a promising plant-based protein source for human nutrition [2,5]. Advances in plant breeding—particularly the development of low-glucosinolate “canola” varieties—and processing have significantly reduced the anti-nutritional factors that previously limited the use of rapeseed proteins in food [5]. Oils extracted from rapeseed varieties are characterised by low glucosinolate (<30 μmol/g) and erucic acid (<2%) levels in the seeds (i.e., ‘double-zero’ rapeseed) [6]. Consequently, scientific research has intensified efforts to incorporate rapeseed meal or its protein isolates into food products and recover high-value compounds from this co-product [7,8]. This reflects a broader trend in sustainable food systems, wherein oilseed byproducts are increasingly regarded as valuable sources of novel protein ingredients and functional components, rather than low-value waste.

One promising approach to rapeseed meal valorisation is the production of bioactive peptides derived from rapeseed proteins. These peptides, which are short protein fragments typically released through enzymatic hydrolysis, have been demonstrated to possess various health-promoting effects. Rapeseed protein hydrolysates are rich in peptides with significant antioxidant properties, including free radical scavenging and metal ion chelation [9]. Furthermore, peptides derived from rapeseeds exhibit antihypertensive effects by inhibiting angiotensin I-converting enzyme (ACE) activity both in vitro and in vivo [9]. Additionally, certain peptides from rapeseed proteins show antidiabetic potential by inhibiting dipeptidyl peptidase IV (DPP-IV), thereby extending the action of incretin hormones [10]. Preliminary investigations have suggested that rapeseed proteins may be a source of antimicrobial peptides [11]. Additional studies have demonstrated that rapeseed peptides can reduce pro-inflammatory cytokine and nitric oxide levels, thereby exerting anti-inflammatory effects [12]. Furthermore, rapeseed-derived peptides have been shown to modulate lipid metabolism, resulting in hypolipidaemic and hypocholesterolemic effects [13]. Notably, certain rapeseed peptides have exhibited anticancer activity in vitro by selectively inhibiting tumour cell proliferation with minimal cytotoxicity toward non-tumour cells [14]. Collectively, these findings highlight the significant nutraceutical and functional potential of rapeseed-derived peptides.

In comparison to other plant protein sources, rapeseed meal holds considerable potential for generating bioactive peptides. Soybean meal (*Glycine max*) remains the most prominent plant-based protein source globally, with around a 40% protein content, and its protein hydrolysates have been extensively studied for nutraceutical peptides [15]. For instance, soy-derived peptides exhibit antioxidant, antihypertensive, hypocholesterolemic, and anticancer activities—a notable example is lunasin, a 43-amino-acid peptide reported to have cholesterol-lowering and anticancer effects [16]. Rapeseed protein, with a comparable protein content (~35–40%) and a well-balanced amino acid profile, has likewise been shown to be of high nutritional quality and similar bioavailability to soy protein in humans [17]. Moreover, rapeseed protein hydrolysates yield a spectrum of bioactive peptides analogous to those from soy—including potent ACE inhibitors, antioxidants, and anti-inflammatory agents—underscoring that rapeseed meal can rival soybean as a source of nutraceutical peptides. Other oilseed meals, such as sunflower and flaxseed, have also been investigated for bioactive peptide production. Sunflower (*Helianthus annuus*) protein hydrolysates, for example, contain peptides with immunomodulatory and anti-inflammatory properties [18]. Nevertheless, soy and rapeseed have generally attracted the greatest research attention in this context, reflecting their higher global production and protein availability.

Papain (EC 3.4.22.2), bromelain (EC 3.4.22.32), and ficin (EC 3.4.22.3) are plant-derived cysteine proteases widely used for the hydrolysis of plant proteins to produce bioactive peptides, serving as valuable tools in the development of functional and nutraceutical protein ingredients [19]. Bromelain, ficin, and papain are cysteine endopeptidases. Bromelain preferentially cleaves peptide bonds adjacent to aromatic, basic, or hydrophobic amino acids; ficin acts on proteins such as casein and gelatin; and papain exhibits broad substrate specificity [20]. These enzymes demonstrate broad substrate specificity and cleave proteins into smaller peptides rather than free amino acids, thus achieving a high degree of hydrolysis and generating low-molecular-weight peptides with diverse bioactive functions [19]. Enzymatic treatment enhances the solubility, digestibility, and overall nutritional quality of plant proteins [21]. Concurrently, they release peptides that exhibit various health-promoting activities, such as antioxidant and antihypertensive effects [21]. As these proteases are plant-derived, their application is consistent with clean-label and vegan product development, facilitating protein hydrolysis without the introduction of animal-derived additives, thereby maintaining the plant-based integrity of the protein ingredient [19,21]. The primary sources of these enzymes are bromelain, ficin, and papain, which are extracted from pineapple, fig latex, and papaya latex, respectively [22].

In silico analysis has become an invaluable method for the efficient utilisation of bioactive peptides derived from rapeseeds. By applying computational tools, researchers can predict the enzymatic cleavage of rapeseed proteins and identify peptide fragments with potential bioactivities prior to empirical experimentation. This methodology utilises databases of known bioactive sequences, such as BIOPEP-UWM, and software that simulates proteolytic processes, thereby facilitating the rapid screening of rapeseed protein sequences for promising peptide motifs [23]. These predictions inform the selection of specific proteases or hydrolysis conditions that optimise the release of peptides with targeted functionalities, such as ACE-inhibitory or antioxidant effects. Rather than relying on trial-and-error methods, researchers can strategically design enzymatic treatments for rapeseed meal, expediting the discovery and development of value-added functional ingredients. Ultimately, the integration of bioinformatics and protein biochemistry facilitates the full exploitation of rapeseed as a source of bioactive peptides.

This study aimed to identify and characterise rapeseed (*Brassica napus* L.) proteins, evaluate their enzymatic hydrolysis in silico using various plant-derived proteases (papain, bromelain, and ficin), and determine the presence of bioactive peptides in the resulting hydrolysates. By comparing the predicted release of functional peptide sequences under different enzymatic treatments, we sought to identify the most effective protease for generating bioactive peptides from rapeseed meal, thereby providing a sustainable approach to valorising this protein-rich co-product of canola oil production.

## 2. Materials and Methods

### 2.1. The Material

The cold-pressed rapeseed meal was provided by AMF Life (Warsaw, Poland). The initial composition of the diet was 34% protein, 3% fat, and 14% dry matter.

### 2.2. The Extraction of Protein from Rapeseed Press Cake

The rapeseed press cake was first ground using a multigrinder (Royal Catering, Berlin, Germany) and then sieved through a sieve (Multiserw LPzE, Marcyporęba, Poland). Particles smaller than 60 mesh (250 µm) were collected for further analysis. The protein extraction procedure involved four main steps: the first extraction, the second extraction, coagulation, and washing of the precipitate.

Then, 400 g of rapeseed flour (particle size <250 µm) was suspended in 1600 g of distilled water and heated to 50 °C on a magnetic stirrer equipped with a temperature sensor (IKA C-MAG HS 7 control, Staufen, Germany). The pH was adjusted to 12.0 by adding NaOH, and the suspension was stirred at this temperature for 60 min to facilitate protein solubilisation. Following extraction, the mixture was centrifuged (MPW-380, Warsaw, Poland) at 3300× *g* for 15 min.

The resulting sediment was re-suspended in distilled water, and the pH was adjusted to 12.0. The suspension was stirred for another 60 min under the same conditions and then centrifuged at 3300× *g* for 15 min. The sediment was discarded, and the supernatants from both extraction steps were combined for coagulation.

The combined supernatant was heated to 50 °C, and its pH was adjusted to 4.5 by adding HCl to induce protein precipitation. The precipitate was separated through centrifugation (3300× *g*, 15 min), and the supernatant was discarded. The sediment was re-suspended in water at a 1:1 (*w*/*w*) ratio without further pH adjustment. Finally, the suspension was subjected to a further centrifugation step (3300× *g*, 15 min), yielding a purified protein pellet.

### 2.3. Electrophoresis

Protein separation was conducted using isoelectric focusing (IEF) followed by SDS-PAGE. Briefly, 50 µg of rapeseed protein was dissolved in 315 µL of rehydration buffer (8 M urea, 4% CHAPS, 70 mM DTT, and 0.5% ampholytes at a pH of 3–10). The samples were then loaded onto 18 cm IPG ReadyStrips (pH 3–10; Bio-Rad, Warsaw, Poland). IEF was performed up to a total of 30 kVh using a PROTEAN IEF system (Bio-Rad). The IPG strips were then equilibrated for 15 min in a buffer containing 50 mM Tris-HCl (pH 8.8), 6 M urea, 30% glycerol, 2% SDS, and 1% DTT and for an additional 15 min in the same buffer supplemented with 2.5% iodoacetamide.

Subsequently, the strips were transferred onto 20 × 20 cm, 1.5 mm thick SDS–polyacrylamide gels (T = 11%, C = 2.6%) prepared according to Laemmli [24]. The second dimension was conducted using a Protean II XI (Bio-Rad, Warsaw, Poland) according to the manufacturer’s instructions. Protein spots were visualised through silver staining using a protocol compatible with mass spectrometry analysis [25].

### 2.4. Protein Spot Excision and Preparation for Mass Spectrometry

Spots of interest were excised from the gels, cut into small fragments, and transferred into 0.5 mL tubes for analysis. The gel pieces were washed three times in 100 µL of 100 mM NH_4_HCO_3_ (pH 8.5) for 5 min, dehydrated with 100 µL of acetonitrile (ACN), and dried in a CentriVap system (Labconco, Warsaw, Poland) at room temperature for 15 min. They were then rehydrated in 100 µL of 10 mM DTT in 50 mM NH_4_HCO_3_ buffer for reduction (56 °C, 60 min). After cooling to room temperature, the solution was replaced with 100 µL of 50 mM iodoacetamide in 50 mM NH_4_HCO_3_, and the samples were incubated in the dark for 45 min at RT. The gel pieces were then washed three times with 100 mL of 100 mM NH_4_HCO_3_ buffer for 5 min at room temperature, dehydrated with 100 µL of ACN, and dried in a CentriVap (Labconco, Warsaw, Poland) for 15 min.

Enzymatic digestion of the proteins was performed on ice through the stepwise addition of 10 µL of 12.5 ng/mL trypsin (Trypsin Gold, mass-spectrometry-grade, Promega, Madison, WI, USA) prepared in 50 mM NH_4_HCO_3_ buffer to ensure complete rehydration of the gel fragments. Subsequently, 30 µL of 50 mM NH_4_HCO_3_ buffer was added to maintain gel hydration overnight at 37 °C. Following digestion, the supernatant was collected, and the peptides were extracted three times using 50 µL of 70% ACN with 1.5% TFA, followed by 15 min of sonication at room temperature in an ultrasonic water bath (Ultron U-507, Ultron, Dywity, Poland). The supernatants were pooled and subsequently dried in a CentriVap system (Labconco, local distributor A.G.A Analytical, Warsaw, Poland) for 45 min at 40 °C.

### 2.5. The MALDI-TOF/TOF Analysis

The peptide pellet was rehydrated in 10 µL of 0.1% TFA and purified using a µC18 ZipTip (Eppendorf, Poznań, Poland) according to the manufacturer’s protocol. A 1 µL aliquot of the purified peptide mixture was mixed with HCCA (with 3,5-dimethoxy-4-hydroxycinnamic acid), spotted onto an AnchoChip frame (Bruker, Poznań, Poland), and then air-dried at room temperature. The mass spectra were acquired using an Ultraflex III MALDI TOF/TOF spectrometer (Bruker, Poznań, Poland) in positive ion reflector mode with an acceleration voltage of 25 kV. External calibration was performed using a peptide calibration standard (Bruker, Poznań, Poland). FlexAnalysis 3.0 software (Bruker-Daltonics) was used to select monoisotopic peptide masses. The identification of proteins and peptides in the mass spectrometry data was performed using the MASCOT algorithm, querying the UniProtKB database (release 2024_04), restricted to the “*Green Plants* (*Viridiplantae*)” taxonomy. The search parameters were set as follows: trypsin as the enzyme, carbamidomethylation of cysteine as a fixed modification, methionine oxidation as a potential modification, and a mass tolerance of 50 ppm.

### 2.6. In Silico Digestion and Bioactivity Prediction Using the BIOPEP Database

The amino acid sequences of the identified proteins were subjected to in silico enzymatic digestion simulations using the BIOPEP database (https://biochemia.uwm.edu.pl/biopep-uwm/ (accessed on 5 April 2025)) [23]. The ‘Enzyme(s) action’ tool in the BIOPEP database was employed to digest each protein using plant-derived enzymes, including papain (EC 3.4.22.2), ficin (EC 3.4.22.3), and stem bromelain (EC 3.4.22.32).

The peptide fragments generated in silico were then analysed using the “search for active fragments” tool in the BIOPEP database. This step facilitated the identification of distinct peptide sequences with documented bioactive properties, providing insights into the potential functions of rapeseed-derived peptides.

### 2.7. Theoretical Prediction of Peptide Bioactivity Using PeptideRanker

All of the peptides produced during the in silico proteolysis outlined in Section 2.6 were subsequently evaluated using PeptideRanker (http://distilldeep.ucd.ie/PeptideRanker/ (accessed on 5 July 2025)). The algorithm assigns each sequence a probability score from 0 to 1; in line with the developers’ guidance, peptides scoring ≥ 0.50 were classified as “bioactive” [26]. This procedure therefore enabled the theoretical determination of which protease generated the greatest number of potentially bioactive peptides.

## 3. Results

### 3.1. Electrophoresis and MALDI-TOF/TOF Protein Identification

**Figure 1:**
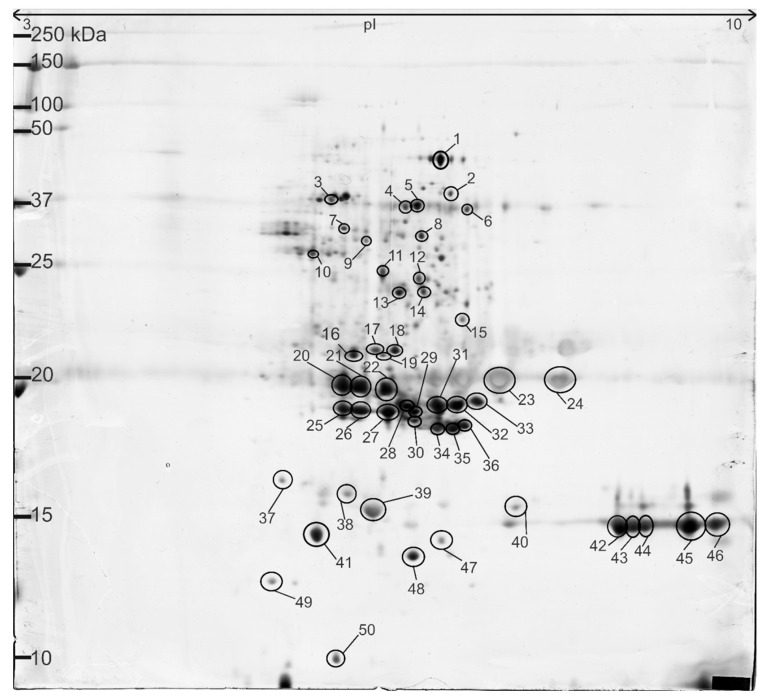
Example of 2D-PAGE gel of rapeseed meal proteins. Proteins were separated in the first dimension on an IPG strip with a pH = 3.0–10.0 and in the second dimension on a 12.5% acrylamide SDS–gel. The numbered spots correspond to the identifications presented in Table 1.

Two-dimensional polyacrylamide gel electrophoresis (2D-PAGE) was used to separate the rapeseed seed protein fractions based on their isoelectric point (a pI range of 3–10) and molecular mass (approximately 10–100 kDa). The resulting gel image (Figure 1) was evaluated qualitatively, so the spot intensities shown are relative rather than quantitative. Fifty of the most intense spots were excised and identified using MALDI-TOF; they are summarised in Table 1.

The table delineates the protein name, UniProt Entry Name, estimated physicochemical parameters (molecular weight and isoelectric point, as determined from the gel), and the species from which the reference sequence was derived, specifically *Brassica napus* or *Arabidopsis thaliana*. The identification of *A. thaliana* proteins among rapeseed proteins is attributable to the close phylogenetic relationship between these two species and their substantial sequence homology. Given that *A. thaliana* is a widely studied model organism for cruciferous plants, its reference protein sequences are extensively represented in databases, frequently resulting in the assignment of homologous *B. napus* proteins to *A. thaliana* [27,28].

The findings revealed that the predominant proteins in the 2D-PAGE profile (26 out of 50 spots) were different forms of cruciferin. Numerous spots (e.g., nos. 4–6, 31–33, 37–46, and 48–50), corresponding to cruciferin subunits (~20–30 kDa), were identified, indicating substantial diversity in isoforms and post-translational modifications. Cruciferin, the major storage protein in *Brassica napus*, is a complex that comprises six monomers. It demonstrates low pH instability in its tertiary structure and exhibits distinct solubility behaviour depending on the pH when intact within the seed cellular matrix [29]. Notably, a vicilin-like protein (a 7S globulin) was identified (spot no. 12; vicilin-like seed storage protein At2g28490) which has been infrequently reported in cruciferous species. In addition to these storage proteins, several metabolic enzymes were detected on the electrophoresis gels. The identified proteins include cytosolic NADP-dependent isocitrate dehydrogenase (spot no. 8), cytoplasmic malate dehydrogenase (spots nos. 13–14), and 3-isopropylmalate dehydrogenase (spot no. 10). Additionally, defence-related proteins, such as myrosinase (spot no. 9) and jacalin-related lectins (JAL36; spots nos. 19, 47), were detected alongside mitochondrial manganese-dependent superoxide dismutase (spots nos. 29–30, 35–36), indicating potential antioxidant functions. Furthermore, oil body proteins were identified, including oil-body-associated protein 1A (spots nos. 20–22 and 25–27), which corresponded to oleosins.

These findings underscore the functional complexity of the protein fraction in rapeseeds, which encompasses storage proteins, metabolic enzymes, defence-related proteins, and oil-body-associated proteins.

### 3.2. In Silico Analysis Using the BIOPEP Database and PeptideRanker

Subsequent to the in silico proteolysis conducted within the BIOPEP database, wherein the protein sequences were enzymatically digested using papain, ficin, and stem bromelain via the “Enzyme(s) action” tool, all resultant peptide fragments were evaluated for bioactive properties through the “search for active fragments” function. Table 2 summarises the total number of peptides generated and those identified as bioactive.

**Figure 2:**
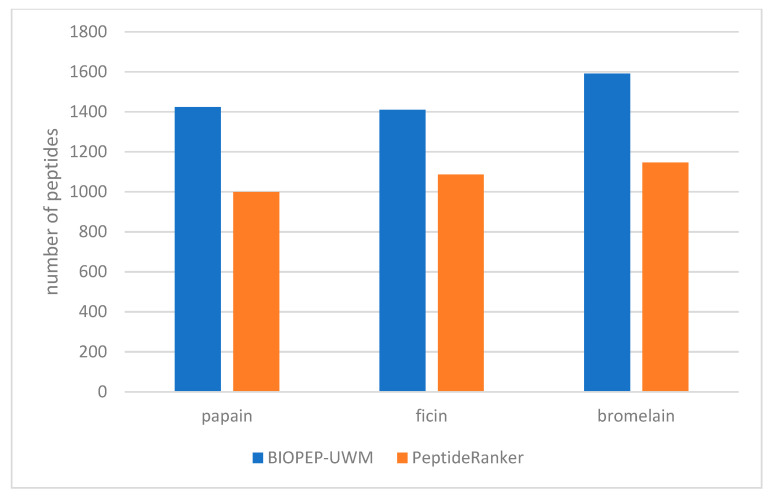
A comparison of the number of potentially bioactive peptides obtained after digestion of identified plant proteins using papain, ficin, and bromelain, predicted using the BIOPEP-UWM “Search for Active Fragments” tool and the PeptideRanker algorithm.

In this study, an in silico methodology was used to assess the bioactive potential of the peptides derived from the proteolytic digestion of selected plant proteins using three plant-derived proteases: papain, bromelain, and ficin. The occurrence of bioactive fragments in each hydrolysate was predicted using two different tools: the BIOPEP-UWM “Search for Active Fragments” module and PeptideRanker. Both predictors agreed that bromelain generated the richest pool of putatively bioactive sequences (Figure 2), identifying 1592 peptides with BIOPEP (Table 2) and 1146 high-scoring peptides with PeptideRanker (Table 3). By comparison, papain produced 1424 (BIOPEP) and 999 (PeptideRanker) putative bioactives, while ficin yielded 1410 and 1086, respectively. Bromelain also produced the highest deterministic counts of total and bioactive peptides; however, as only single simulations were performed, this difference was not tested for statistical significance.

The resulting hydrolysates exhibited a remarkably extensive range of peptide biological activities, with 39 distinct types of bioactivities identified through the analysis in the BIOPEP-UWM “Search for Active Fragments” module (Table 4). On average, each hydrolysate contained peptides with 15–17 biological functions. Even the substrate that produced the fewest peptides (36 peptides from OBP1A_ARATH digested by papain) exhibited as many as 11 distinct activities, including a unique anticancer property specific to this protein. In contrast, the bromelain-digested BGL19_ARATH hydrolysate displayed the highest functional diversity, with 23 different bioactivities among the 116 identified peptides. These findings suggest a positive correlation between the total number of released peptides and the diversity of their biological functions. Larger proteins that yield more fragments tend to exhibit a broader spectrum of potential health-promoting effects.

**Figure 3:**
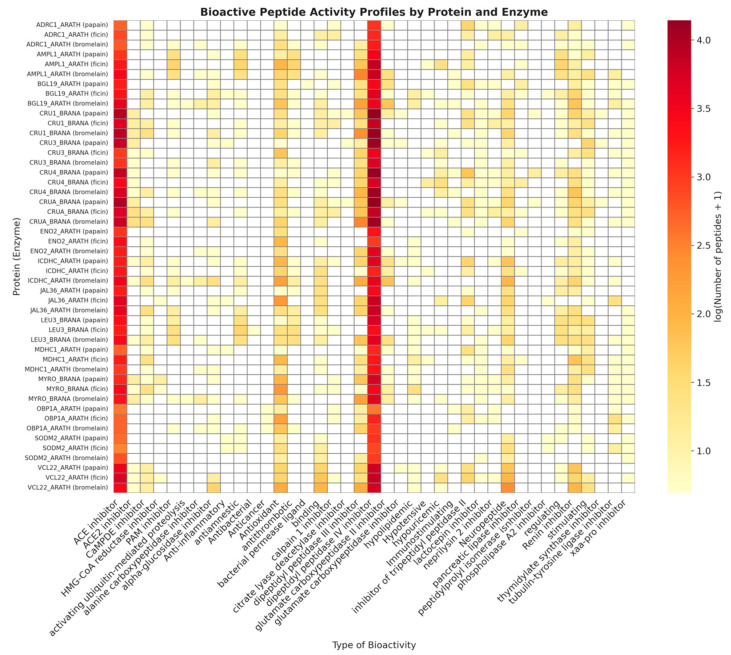
A heatmap illustrating the predicted bioactive peptide activity profiles resulting from the in silico enzymatic hydrolysis of identified rapeseed proteins.

Among the hydrolysates analysed, the peptides most frequently detected were those that inhibited angiotensin I-converting enzyme (ACE), blocked dipeptidyl peptidase IV (DPP-IV), or exhibited antioxidant properties; these activities were present in 100% of the samples, regardless of the protease employed (Figure 3). Collectively, these peptides represent the most abundant group of bioactive molecules in the human body. For example, the bromelain-derived CRU1_BRANA hydrolysate yielded 149 identified peptides, of which 60 inhibited DPP-IV, 49 inhibited ACE, and 4 demonstrated antioxidant properties. Similarly, the papain-derived CRUA_BRANA hydrolysate produced 151 peptides, of which 62, 52, and 3 exhibited the aforementioned activities. Additionally, renin inhibitors and neuropeptides were frequently observed, along with DPP-III inhibitors and peptides characterised as “stimulating” or “binding”.

Infrequent but potentially significant biological functions were observed sporadically. Anticancer activity was identified exclusively in the hydrolysates of OBP1A_ARATH, where each of the three enzymes—papain, ficin, and bromelain—released precisely one peptide exhibiting this activity. Antibacterial activity was similarly rare, represented by a single peptide in the ficin-digested LEU3_BRANA hydrolysate. Other peptides exerting effects on bacterial cells through alternative mechanisms, such as a permease ligand identified solely in the papain-digested BGL19_ARATH hydrolysate, were infrequently observed. Furthermore, only a limited number of hydrolysates contained peptides that inhibited critical enzymes, including thymidylate synthase (essential for DNA synthesis and a potential anticancer target), HMG-CoA reductase (involved in cholesterol metabolism), and phospholipase A_2_ (which may have anti-inflammatory effects). Although only a few of these specialised peptides were identified (1–2 per hydrolysate), their presence underscores the potential to obtain not only major functional groups but also less common yet biologically significant peptides from the investigated plant proteins.

## 4. Discussion

Cruciferin, the predominant storage protein in rapeseeds, is classified within the 11S globulin family and constitutes approximately 60% of the total seed protein content [30]. This study identified multiple spots corresponding to various cruciferin isoforms, such as CRU1, CRU4, and CRUA, catalogued in the UniProt database. Structurally, cruciferin is a hexamer consisting of six subunits with a molecular weight of approximately 300–360 kDa. It is composed of pairs of polypeptides, a heavier α chain (~30 kDa) and a lighter β chain (~20 kDa), which are linked by a disulfide bond [31,32]. Under 2D-PAGE conditions, this complex dissociates into its subunits, resulting in numerous spots in the ~20–30 kDa range, corresponding to individual α/β chains and their post-translational modifications, as noted by Nietzel et al. [33]. Rapeseed contains multiple forms of cruciferin, with over 30 distinct variants identified, including various α and β isoforms and post-translational modifications. Gu et al. [34] observed multiple spots corresponding to individual α/β subunits and their modifications, further highlighting the extensive heterogeneity of this storage protein family.

Napin (2S albumin, ~17 kDa) is the second most abundant storage protein in rapeseeds, constituting approximately 20% of the total protein content in canola seeds [31,32]. Notably, typical napin spots were not observed on the two-dimensional gel. This absence may be attributed to methodological limitations, as napin is highly basic (pI ~10.5) [35], potentially placing it outside of the pH 3–10 gradient range used during isoelectric focusing.

Notably, one location within the analysed material was identified as a vicilin-like protein, which is a member of the 7S globulin family. Vicilins are predominantly associated with leguminous plants and are infrequently documented in cruciferous species [36]. Nevertheless, recent proteomic studies have confirmed the presence of vicilin-like proteins in *Brassica rapa* seeds [37]. Consequently, the detection of a vicilin-like protein in rapeseed meal implies that 7S globulins may also exist at trace levels in this crop.

Among the metabolic enzymes identified on the two-dimensional gel were those associated with the tricarboxylic acid cycle (the Krebs cycle), specifically cytosolic NADP-dependent isocitrate dehydrogenase and cytoplasmic malate dehydrogenase. Additionally, 3-isopropylmalate dehydrogenase, which is involved in the biosynthesis of branched-chain amino acids, was identified. The presence of these dehydrogenase enzymes in seeds was substantiated by Gu et al. [34] in their study on the protein expression during rapeseed germination.

Rapeseed seeds contain defence proteins that protect them against pests and pathogens. Among these proteins is myrosinase, a thioglucosidase enzyme that catalyses the hydrolysis of glucosinolates into toxic compounds, such as isothiocyanates, upon tissue damage [38]. Thangstad et al. [39] have reported that myrosinases represent a group of isoenzymes found in Brassicaceae species, including *Brassica napus*. Similarly, Geshi et al. [40] confirmed the presence of multiple myrosinase isoforms in Brassica species.

The jacalin-related lectin family is another crucial component of plant defence mechanisms. These lectins, induced in response to stress and damage, play essential roles in plant immunity [41]. Supporting this notion, Gu et al. [34] identified a jacalin-like lectin protein in their two-dimensional gels of germination-related proteins, underscoring the importance of these lectins to the defence mechanisms of rapeseed.

Rapeseed meal is also characterised by the presence of enzymes that alleviate oxidative stress and its derivatives. Notably, mitochondrial manganese-dependent superoxide dismutase acts as the primary defence mechanism against reactive oxygen species by scavenging superoxide radicals, thereby safeguarding seed cells from oxidative damage [42].

The protein profile of rapeseed meal, as elucidated through 2D-PAGE, revealed a complex composition predominantly characterised by the storage protein cruciferin, alongside metabolic and defensive enzymes typical of canola seeds [43]. This profile aligns with the established rapeseed seed proteome; however, the identification of a vicilin-like protein presents an intriguing opportunity for further research.

In silico digestion of rapeseed proteins identified in this study, followed by an analysis of the resultant bioactive peptides, demonstrated a diverse array of potential activities, including the inhibition of ACE and DPP-IV, as well as antioxidant, antibacterial, and anticancer properties. Studies with the three plant cysteine proteases showed that bromelain released the highest number of peptides overall—including the greatest number of putatively bioactive sequences—compared with papain and ficin—including putatively bioactive sequences—when compared with papain and ficin. The higher number of bioactive peptides generated during bromelain hydrolysis can be explained by the complementarity between its substrate specificity and the amino acid composition of the major rapeseed proteins. Bromelain preferentially cleaves the peptide bonds adjacent to lysine, tyrosine, and hydrophobic residues [44], which are abundantly present in the sequence of cruciferin, the primary storage protein of rapeseed. This alignment between the enzyme’s cleavage preferences and the distribution of residues in rapeseed proteins results in a greater number of accessible cleavage sites than those available to ficin, which favours arginine at the P_1_ position, or papain, which targets predominantly hydrophobic motifs [21]. Consequently, bromelain seems to generate more short peptides with potential biological activity. The subsequent analysis of these peptides indicated a diverse range of predicted functions, including inhibition of angiotensin-converting enzyme (ACE) and dipeptidyl peptidase-IV (DPP-IV), alongside antioxidant, antibacterial and anticancer properties.

Notably, the predominant category of identified peptides consisted of those with potential antihypertensive and antidiabetic properties, as enzymatic hydrolysis liberated numerous ACE- and DPP-IV-inhibitory peptides from each of the analysed proteins. Angiotensin-converting enzyme (ACE) and dipeptidyl peptidase IV (DPP-IV) are integral to the pathogenesis of hypertension and type 2 diabetes mellitus, respectively. Consequently, the inhibition of ACE and DPP-IV by specific peptides has emerged as a novel therapeutic approach to complementing the pharmacological treatments for both conditions [45]. In their investigation of oilseed plants, Han et al. [45] digested rapeseed cruciferin and napin in silico using pepsin. Their findings indicate that oilseed proteins, including those derived from rapeseed, are promising sources of peptides with ACE- and DPP-IV-inhibitory activities, underscoring their potential utility in the management of hypertension and diabetes. Similarly, Duan et al. [46] employed bioinformatic methodologies to assess the bioactive peptides within rapeseed protein. These results corroborate the aforementioned findings, demonstrating that rapeseed protein holds significant potential for generating various biologically active peptides, particularly those with DPP-IV- and ACE-inhibitory properties. Furthermore, You et al. [47] simulated gastrointestinal digestion of rapeseed proteins and subsequently isolated the most promising DPP-IV-inhibitory peptides. These peptides were synthesised and confirmed to inhibit DPP-IV activity in vitro, underscoring rapeseed protein hydrolysates as a rich source of peptides with potential antidiabetic effects, as predicted by bioinformatic analyses and verified in experimental studies. Recent in vivo studies support the antihypertensive and antidiabetic potential of rapeseed-protein-derived peptides. In spontaneously hypertensive rats, oral administration of these peptides reduced their systolic blood pressure, with a more pronounced and prolonged effect when combined with captopril. This synergistic action was not associated with additional ACE inhibition but likely involved enhanced nitric oxide production and endothelial function [48]. In type 2 diabetic mouse models, specific hydrolysates (RCPP-3, RNPP-1) improved glycaemic control and lipid metabolism through the stimulation of GLP-1 secretion via the activation of the intestinal calcium-sensing receptors and subsequent PI3K/Akt pathway activation [49]. Encapsulation of the peptides into biopolymeric nanoparticles further enhanced their hypoglycaemic effect, indicating improved gastrointestinal stability and bioavailability [50].

Among the identified peptides, several exhibited uncommon biological activities, including potential anticancer properties. Ma et al. [51] investigated a peptide derived from rapeseed proteins for its effects on HepG2 liver cancer cells. Their findings demonstrated that this peptide significantly inhibited cancer cell proliferation, suggesting that enzymatic hydrolysis of rapeseed proteins may yield peptides with antitumour activities. Ferrero et al. [52] reported the presence of polypeptides and oligosaccharides in enzymatic hydrolysates of rapeseed proteins that inhibited the growth of the MCF-7 breast cancer cell line with moderate cytotoxicity to human cells. In a murine breast cancer model, nanocomplexes releasing pro-apoptotic peptides in response to tumour-associated cathepsin B significantly improved the efficacy of doxorubicin, reducing tumour growth by up to 91% [53]. This indicates the potential application of these hydrolysates in cancer treatment; however, further research is required.

Although our study identified only one antibacterial peptide, the existing literature indicates that rapeseed is a promising source of antimicrobial peptides. For example, Duan et al. [11] utilised an in silico approach to generating antibacterial peptides from rapeseed proteins. Additionally, Rahman et al. [54] discussed the antimicrobial properties of rapeseed storage proteins, suggesting a correlation between their spatial structure and antimicrobial efficacy, and confirmed that these proteins retained their properties following enzymatic hydrolysis. Through digestion simulation using various enzymes, 26 peptide sequences were identified to have potential antimicrobial activity, with trypsin proteolysis producing the greatest number of candidates. These findings suggest that rapeseed seed proteins are a theoretically abundant source of antimicrobial peptides, meriting further empirical investigation.

Additional peptide activities predicted in our in silico analysis have been substantiated by the existing literature. For example, He et al. [55] validated the antioxidant properties of rapeseed-derived peptides produced via fermentation or sequential enzymatic hydrolysis. Similarly, Yang et al. [56] identified rapeseed peptides with significant hypolipidaemic effects.

Our findings align with the existing literature, corroborating the significant potential of rapeseed proteins as a source of bioactive peptides. However, it is important to note that in silico predictions, while instrumental in guiding experimental design, do not replicate the actual enzymatic conditions encountered in the laboratory. Tools such as BIOPEP-UWM perform “virtual” proteolysis under the simplifying assumption of ideal and complete hydrolysis—every peptide bond that matches the stored recognition motif of a selected protease is cleaved [23]. In practice, hydrolysis is rarely exhaustive; the steric hindrance imposed by the native tertiary/quaternary structure of plant proteins; competition between multiple cleavage sites; and incomplete enzyme–substrate contact can all prevent the formation of some of the theoretically predicted peptides, generating false positive results. Conversely, the specificity matrices embedded into BIOPEP-UWM are compiled from a finite set of experimentally verified cleavage sites; any gaps in this knowledge may lead to false negative omissions, particularly for less studied plant proteases or storage proteins rich in disulfide-bonded domains [23]. Moreover, the current algorithms do not model the kinetic or environmental parameters—enzyme concentration, reaction time, pH, temperature, ionic strength, or post-translational modifications—so they yield only a qualitative inventory of putative fragments rather than a quantitative profile of the peptide release. Variables such as enzyme specificity, substrate interactions, and the presence of other macromolecules can influence peptide release and functionality. An additional layer of uncertainty is introduced when the predictions are ranked using PeptideRanker, which assigns probabilistic bioactivity scores based solely on primary sequence features learned from known peptides; this approach may miss novel activities outside of the training set and produce high-scoring false positives if contextual factors such as the peptide conformation, stability, or bioavailability are unfavourable in vivo [26]. Because PeptideRanker does not distinguish between different modes of action and relies on a user-defined score threshold, its output should likewise be interpreted as a prioritisation aid rather than definitive evidence of bioactivity. In accordance with the above considerations and the recent literature [57,58], in silico predictions should be regarded as an efficient preliminary screening approach to prioritising experimental targets; however, they are not sufficient to substantiate biological activity without subsequent in vitro and in vivo validation.

The in vivo efficacy of the rapeseed-derived peptides predicted herein is contingent on their bioaccessibility within the gastrointestinal milieu, their resistance to peptidases, and their subsequent transepithelial transport in an intact, biologically active form [59]. Although many oligopeptides are rapidly hydrolysed or display limited paracellular permeability, certain di- and tripeptides can cross the enterocyte via the PepT1 transporter and enter the bloodstream intact [60]. Key physicochemical parameters—such as molecular mass, charge, and hydrophobicity—govern these processes; hence, simulated gastrointestinal digestion followed by Caco-2 monolayer assays constitutes a critical next step in ranking peptides [61]. From a toxicological standpoint, peptides derived from edible proteins are generally regarded as low-risk. Nevertheless, every novel fraction must be rigorously screened for both allergenicity and cytotoxicity. In the study by Ferrero et al. mentioned earlier [52], rapeseed protein hydrolysates exhibited marked antiproliferative activity against tumour cell lines while causing only marginal cytotoxicity in normal human fibroblasts, indicating a favourable preliminary safety window. Despite these promising results, exhaustive in vitro and in vivo investigations remain essential before these bioactive peptides can be responsibly integrated into functional foods or nutraceutical formulations.

## 5. Conclusions

The findings of this study substantiate that rapeseed meal is a promising source of bioactive peptides. The proteomic analysis has identified cruciferin as the predominant storage protein. In silico digestion using papain, ficin, and bromelain released a variety of peptides exhibiting ACE-inhibitory, DPP-IV-inhibitory, and antioxidant activities. Bromelain generated the largest number of putative bioactive peptides in our single-run simulations; future replicated studies are required to confirm whether this difference is statistically significant. While these results highlight the nutraceutical value of rapeseed meal, further in vitro and in vivo investigations are required to validate the predicted health benefits of these peptides in vivo.

## Figures and Tables

**Table 1:** Proteins identified from rapeseed meal using MALDI-TOF.

Spot ID	Protein Name	UniProt Entry Name	Species	Mass	pI
1	Beta-glucosidase 19	BGL19_ARATH	*Arabidopsis thaliana*	45.3	6.90
2	Cruciferin CRU4	CRU4_BRANA	*Brassica napus*	37.3	6.94
3	Leucine aminopeptidase 1	AMPL1_ARATH	*Arabidopsis thaliana*	36.7	5.84
4	Cruciferin CRU4	CRU4_BRANA	*Brassica napus*	37.0	6.67
5	Cruciferin CRU4	CRU4_BRANA	*Brassica napus*	37.0	6.70
6	Cruciferin CRU4	CRU4_BRANA	*Brassica napus*	34.9	7.17
7	Bifunctional enolase 2/transcriptional activator	ENO2_ARATH	*Arabidopsis thaliana*	31.3	5.95
8	Cytosolic isocitrate dehydrogenase [NADP]	ICDHC_ARATH	*Arabidopsis thaliana*	30.9	6.71
9	Myrosinase	MYRO_BRANA	*Brassica napus*	29.2	6.12
10	3-isopropylmalate dehydrogenase, chloroplastic	LEU3_BRANA	*Brassica napus*	28.6	5.64
11	Cruciferin CRU4	CRU4_BRANA	*Brassica napus*	24.9	6.33
12	Vicilin-like seed storage protein At2g28490	VCL22_ARATH	*Arabidopsis thaliana*	24.7	6.69
13	Malate dehydrogenase 1, cytoplasmic	MDHC1_ARATH	*Arabidopsis thaliana*	24.9	6.50
14	Malate dehydrogenase 1, cytoplasmic	MDHC1_ARATH	*Arabidopsis thaliana*	24.6	6.70
15	Cruciferin CRU1	CRU3_BRANA	*Brassica napus*	21.3	7.21
16	NADPH-dependent aldehyde reductase 1, chloroplastic	ADRC1_ARATH	*Arabidopsis thaliana*	20.4	6.10
17	NADPH-dependent aldehyde reductase 1, chloroplastic	ADRC1_ARATH	*Arabidopsis thaliana*	20.3	6.32
18	NADPH-dependent aldehyde reductase 1, chloroplastic	ADRC1_ARATH	*Arabidopsis thaliana*	20.4	6.48
19	Jacalin-related lectin 36	JAL36_ARATH	*Arabidopsis thaliana*	20.2	6.38
20	Oil-body-associated protein 1A	OBP1A_ARATH	*Arabidopsis thaliana*	20.4	5.97
21	Oil-body-associated protein 1A	OBP1A_ARATH	*Arabidopsis thaliana*	20.1	6.18
22	Oil-body-associated protein 1A	OBP1A_ARATH	*Arabidopsis thaliana*	19.8	6.42
23	Cruciferin CRU4	CRU4_BRANA	*Brassica napus*	20.2	7.56
24	Cruciferin CRU4	CRU4_BRANA	*Brassica napus*	20.1	8.15
25	Oil-body-associated protein 1A	OBP1A_ARATH	*Arabidopsis thaliana*	17.6	5.97
26	Oil-body-associated protein 1A	OBP1A_ARATH	*Arabidopsis thaliana*	17.5	6.06
27	Oil-body-associated protein 1A	OBP1A_ARATH	*Arabidopsis thalian*	17.8	6.43
28	Cruciferin CRU4	CRU4_BRANA	*Brassica napus*	17.8	6.64
29	Superoxide dismutase [Mn] 2, mitochondrial	SODM2_ARATH	*Arabidopsis thaliana*	17.7	6.68
30	Superoxide dismutase [Mn] 2, mitochondrial	SODM2_ARATH	*Arabidopsis thaliana*	17.4	6.67
31	Cruciferin	CRUA_BRANA	*Brassica napus*	17.7	6.92
32	Cruciferin	CRUA_BRANA	*Brassica napus*	17.7	7.09
33	Cruciferin	CRUA_BRANA	*Brassica napus*	17.9	7.27
34	Cruciferin CRU4	CRU4_BRANA	*Brassica napus*	17.0	6.92
35	Superoxide dismutase [Mn] 2, mitochondrial	SODM2_ARATH	*Arabidopsis thaliana*	17.0	7.09
36	Superoxide dismutase [Mn] 2, mitochondrial	SODM2_ARATH	*Arabidopsis thaliana*	17.0	7.14
37	Cruciferin BnC1	CRU1_BRANA	*Brassica napus*	16.0	5.30
38	Cruciferin	CRUA_BRANA	*Brassica napus*	15.9	6.00
39	Cruciferin	CRUA_BRANA	*Brassica napus*	15.1	6.25
40	Cruciferin CRU4	CRU4_BRANA	*Brassica napus*	15.1	7.81
41	Cruciferin CRU4	CRU4_BRANA	*Brassica napus*	13.5	5.70
42	Cruciferin CRU4	CRU4_BRANA	*Brassica napus*	13.6	8.74
43	Cruciferin CRU4	CRU4_BRANA	*Brassica napus*	13.6	8.95
44	Cruciferin CRU4	CRU4_BRANA	*Brassica napus*	13.6	9.98
45	Cruciferin CRU4	CRU4_BRANA	*Brassica napus*	13.6	9.44
46	Cruciferin CRU4	CRU4_BRANA	*Brassica napus*	13.6	9.70
47	Jacalin-related lectin 36	JAL36_ARATH	*Arabidopsis thaliana*	13.3	7.07
48	Cruciferin CRU4	CRU4_BRANA	*Brassica napus*	13.0	6.69
49	Cruciferin CRU4	CRU4_BRANA	*Brassica napus*	12.4	5.29
50	Cruciferin BnC1	CRU1_BRANA	*Brassica napus*	10.1	5.97

**Table 2:** Total number of peptides and bioactive peptides generated through in silico digestion of proteins with papain, ficin, and bromelain using BIOPEP database.

Protein Name	Papain Digestion		Ficin Digestion		Bromelain Digestion	
	Total Peptides	Bioactive Peptides	Total Peptides	Bioactive Peptides	Total Peptides	Bioactive Peptides
ADRC1_ARATH	139	49	143	55	149	57
AMPL1_ARATH	164	75	174	107	188	119
BGL19_ARATH	176	80	198	95	199	116
CRU1_BRANA	211	150	213	115	216	149
CRU3_BRANA	215	125	218	76	222	77
CRU4_BRANA	197	142	204	98	207	133
CRUA_BRANA	211	151	213	123	216	141
ENO2_ARATH	164	53	168	60	174	76
ICDHC_ARATH	147	102	152	73	170	121
JAL36_ARATH	176	70	193	117	199	110
LEU3_BRANA	173	92	171	86	183	110
MDHC1_ARATH	131	52	136	80	149	66
MYRO_BRANA	166	93	182	94	183	98
OBP1A_ARATH	83	36	88	59	91	53
SODM2_ARATH	87	44	97	43	103	45
VCL22_ARATH	160	110	174	129	180	121
in total	2600	1424	2724	1410	2829	1592

**Table 3:** Number of PeptideRanker-predicted bioactive peptides (score ≥ 0.50) generated through in silico digestion of rapeseed proteins with papain, ficin, and bromelain.

Protein	Papain Digestion	Ficin Digestion	Bromelain Digestion
ADRC1_ARATH	51	42	53
AMPL1_ARATH	70	45	102
BGL19_ARATH	52	96	41
CRU1_BRANA	77	79	76
CRU3_BRANA	58	69	96
CRU4_BRANA	85	81	91
CRUA_BRANA	63	78	74
ENO2_ARATH	48	58	49
ICDHC_ARATH	59	67	55
JAL36_ARATH	58	66	45
LEU3_BRANA	63	66	91
MDHC1_ARATH	36	35	53
MYRO_BRANA	98	111	112
OBP1A_ARATH	36	41	39
SODM2_ARATH	30	34	38
VCL22_ARATH	115	118	131
In total	999	1086	1146

**Table 4:** Predicted bioactive peptide activities resulting from in silico enzymatic hydrolysis of identified rapeseed proteins using papain, ficin, and bromelain, showing the number of peptide sequences associated with each predicted activity and the total number of peptides generated by each enzyme.

Bioactivity	Papain Digestion	Ficin Digestion	Bromelain Digestion
ACE inhibitor	446	428	464
ACE2 inhibitor	11	9	12
activating ubiquitin-mediated proteolysis	0	0	3
alanine carboxypeptidase inhibitor	0	0	13
alpha-glucosidase inhibitor	9	6	22
antiamnestic	10	13	17
antibacterial	0	1	0
anticancer	1	1	1
anti-inflammatory	3	3	0
antioxidative	40	74	63
antithrombotic	8	11	17
bacterial permease ligand	1	0	0
binding	15	25	26
calpain I inhibitor	8	11	0
CaMPDE inhibitor	9	19	25
citrate lyase deacetylase inhibitor	3	0	0
dipeptidyl peptidase III inhibitor	38	38	94
dipeptidyl peptidase IV inhibitor	597	528	610
glutamate carboxypeptidase II inhibitor	14	5	23
glutamate carboxypeptidase inhibitor	5	0	0
HMG-CoA reductase inhibitor	2	3	0
hypolipidemic	5	10	8
hypotensive	0	9	0
hypouricemic	4	16	6
immunostimulating	3	0	6
inhibitor of tripeptidyl peptidase II	37	29	0
lactocepin inhibitor	8	8	11
neprilysin 2 inhibitor	8	11	0
neuropeptide	32	33	50
PAM inhibitor	8	11	17
pancreatic lipase inhibitor	5	8	1
peptidylprolyl isomerase inhibitor	4	0	0
phospholipase A2 inhibitor	1	3	0
regulating	16	18	17
renin inhibitor	34	36	42
stimulating	21	21	20
thymidylate synthase inhibitor	3	0	0
tubulin-tyrosine ligase inhibitor	5	12	12
xaa-pro inhibitor	10	10	11
Total peptides	1424	1410	1592

## Data Availability

The original contributions presented in the study are included in the article, further inquiries can be directed to the corresponding author.
